# Accumulation and Subcellular Distribution Patterns of Carbamazepine in Hydroponic Vegetables

**DOI:** 10.3390/biology14040343

**Published:** 2025-03-26

**Authors:** Sihan Yao, Yan Chen, Nan Zheng, Ting Chen, Sufen Zhang, Zhiyang Yu, Haiyan Wang

**Affiliations:** Key Laboratory of Nuclear Agricultural Sciences of Ministry of Agriculture, Institute of Nuclear Agricultural Sciences, College of Agriculture and Biotechnology, Zhejiang University, Hangzhou 310058, China; 22216008@zju.edu.cn (S.Y.); chenchunyue@zju.edu.cn (Y.C.); 3200101630@zju.edu.cn (N.Z.); 12016001@zju.edu.cn (T.C.); sfzhang@zju.edu.cn (S.Z.); yuzhiyang@zju.edu.cn (Z.Y.)

**Keywords:** carbamazepine, ^14^C isotope tracer technology, accumulation, subcellular distribution, affecting factors

## Abstract

Pharmaceutical and Personal Care Products (PPCPs), such as carbamazepine, enter the food chain through wastewater irrigation, posing risks to ecosystems and human health. However, research on the translocation and subcellular distribution of carbamazepine in vegetables is limited. Herein, we used ^14^C-labeled carbamazepine as a tracer to investigate its removal, accumulation, and subcellular compartmentalization in hydroponic vegetable systems. Results showed carbamazepine accumulated in Chinese flowering cabbage and water spinach with removal efficiencies of 93.0–93.2%. The compound was absorbed by roots and translocated to aboveground tissues, particularly in bottom leaves, reaching 90.3 μmol/kg after 768 h, as confirmed by autoradiography. Subcellular analysis indicated that carbamazepine is predominantly distributed in root organelles and in the soluble fraction of leaves and stems. A strong correlation (R^2^ > 0.800) was observed between root enrichment coefficients and log *K*_OW_ for caffeine, carbamazepine, and kresoxim-methyl. Higher lipid content in water spinach roots (2.07%) significantly enhanced upward transport, underscoring lipid content’s role in translocation. Additionally, a higher xylem content in the plant accelerated the transport of carbamazepine. This study provides key insights into the environmental behavior of organic pollutants, supporting efforts in environmental and health protection.

## Simple Summary

In many places, using wastewater for irrigation allows medicines like carbamazepine to enter our food supply. This study looked at how this common drug, used to treat certain health conditions, is taken up by two leafy vegetables grown in water. We used a special marking method to track carbamazepine’s fate from the water into the plants. We found that both vegetables removed over 90% of carbamazepine, which built up mostly in the lower leaves. Subcellular analysis showed that carbamazepine mainly accumulates in organelles within the roots and in the watery parts of leaves and stems. We also discovered that how quickly the drug moves depends on its chemical features and the amount of fats in the roots. In one plant, higher fat content and more water-carrying tissues led to faster transport. These findings help us understand how medicines can end up in our food and highlight the need to manage wastewater carefully to protect both people and the environment.

## 1. Introduction

Pharmaceutical and Personal Care Products (PPCPs) are widely detectable in water environments around the world, including surface water for agricultural irrigation, urban and forest groundwater, and treated drinking water [1,2,3]. According to the European Environment Agency, only 40% of Europe’s natural water bodies (lakes, rivers, and coastal waters) are in good ecological health, with unregulated human activities accelerating their degradation [4]. In a study by Williams et al. [5], concentrations of carbamazepine, antibiotics, and nonsteroidal anti-inflammatory drugs reached as high as 1900 ng/L in untreated wastewater entering India’s Ahar River, significantly exceeding levels of other pollutants. Conventional wastewater treatment processes are unable to fully remove organic pollutants [6], allowing these compounds to persist in treated water, which is then used for agricultural irrigation [7]. Such water can be absorbed by plants, where pollutants accumulate and eventually enter the food chain [8]. Given the widespread distribution and persistence of many PPCPs, including those that disrupt endocrine systems [9], it is critical to investigate their environmental distribution, transport, and accumulation.

Carbamazepine (CBZ; its physicochemical properties are listed in Table S1), a medication used to treat epilepsy and neuralgic pain, has been detected in wastewater effluents, sludge, and soil at concentrations ranging from 0.24 to 2.1 μg/L, 7.8 to 258 μg/kg, and 0.0065 to 7.5 μg/kg, respectively [10]. Wastewater treatment plants typically remove less than 10% of carbamazepine, and its degradation half-life in both plants and soil exceeds 100 days [11,12]. In aerobic soil, the degradation half-life of carbamazepine is over 120 days, posing a high risk of accumulation in plants [13]. Ecotoxicological studies have also highlighted carbamazepine’s acute toxicity at environmental concentrations [14,15]. Its persistence in wastewater and biosolids increases the risk of bioaccumulation [16], emphasizing the importance of studying carbamazepine within water-vegetable systems.

PPCPs display a wide range of physicochemical properties, from highly hydrophilic (e.g., caffeine, log *K*_OW_ = −0.07; atenolol, log *K*_OW_ = 0.16) to highly hydrophobic (e.g., atorvastatin, log *K*_OW_ = 6.36; triclocarban, log *K*_OW_ = 4.90). Characteristics such as hydrophobicity and ionization, along with biological factors like plant transpiration, critically affect pollutant absorption and transport in plants [17,18,19,20]. Different plants exhibit varying capacities to remove pollutants. For instance, yellow flag iris removed 87% of codeine from water within 48 h, while water pennywort, Vietnamese coriander, and water hyacinth achieved removal rates below 33% under the same conditions [21]. However, many studies have limited focus on how the physicochemical properties of pollutants, plant traits, and exposure duration influence pollutant absorption and transport. Understanding these interactions is vital for evaluating plant the uptake and transport of organic pollutants.

This study investigates the uptake, transport, and targeted accumulation of carbamazepine in a hydroponic-vegetable system (Chinese flowering cabbage and water spinach) using ^14^C-labeled carbamazepine as a tracer. This study also explores the subcellular compartmentalization of carbamazepine in Chinese flowering cabbage and examines the influence of physicochemical properties of organic pollutants (e.g., hydrophobicity), biological characteristics of plants (e.g., root lipid content), and exposure duration on uptake and transport of pollutants. The findings provide a scientific basis for assessing the environmental safety of carbamazepine and offer technical and data support for future research on the environmental behavior and risk assessment of other organic pollutants.

## 2. Materials and Methods

### 2.1. Chemicals

The ^14^C-labeled carbamazepine was purchased from American Radiolabeled Chemicals, Inc. (St. Louis, MO, USA), with a chemical and radiochemical purity greater than 97% and a specific radioactivity of 50.0 mCi/mmol. Analytical grade reagents, including methanol, glacial acetic acid, ethylene glycol ether, xylene, ethanolamine, sucrose, and petroleum ether, were purchased from Sinopharm Chemical Reagent Co., Ltd. (Shanghai, China). Tris-HCl was purchased from Solarbio Science & Technology Co., Ltd. (Beijing, China), and dithiothreitol was sourced from Macklin Biochemical Co., Ltd. (Shanghai, China). The scintillation reagents 2,5-diphenyloxazole (PPO) and 1,4-di-[2-(5-phenyloxazol)]-benzene (POPOP) were purchased from TCI Co., Ltd. (Shanghai, China). Scintillation cocktail I consisted of 0.5 g POPOP, 7 g PPO, 350 mL ethylene glycol ether, and 650 mL xylene. Scintillation cocktail II consisted of 0.5 g POPOP, 7 g PPO, 225 mL ethylene glycol ether, and 600 mL xylene, and after dissolving, 175 mL ethanolamine was added. The nutrient solution consisted of standard Hoagland’s formulation, with pH adjusted to 6.5. Subcellular fractionation buffer was composed of 50 mmol/L Tris-HCl (pH 7.5), 250 mmol/L sucrose, and 1.0 mmol/L dithiothreitol.

### 2.2. Plant Cultivation

Chinese flowering cabbage (*Brassica campestris* ssp. *chinensis* var. parachinensis ‘Youqing 49’) and water spinach (*Ipomoea aquatica* Forssk.) were used as test plants, with seeds purchased from the Shiqi Urban Horticulture Company (Suqian, China). Chinese flowering cabbage seeds were soaked in 40 °C water for 4 h, and water spinach seeds for 12 h. After soaking, seeds were placed on moistened gauze in petri dishes and kept in the dark until germination. Germinated seeds were transplanted into seedling trays filled with soil and grown under controlled conditions (80% humidity, 16 h light, 25 °C/18 °C day/night) until the plants developed 3-4 true leaves (Figure S1). Uniform plants with good growth were selected, and roots were disinfected in 0.1% potassium permanganate before being transferred to hydroponic systems with Hoagland’s nutrient solution in brown glass bottles wrapped in aluminum foil to prevent light exposure.

### 2.3. Plant Uptake and Transport Experiment

After one week of hydroponic acclimatization, the plants were exposed to carbamazepine (0.03 μCi, 1 mg/L) in 30 mL nutrient solution. During the experiment, nutrient solution without pollutants was added to the bottles daily to maintain a volume of 30 mL (Figure S2). Sampling points were set at 0.25 d, 0.5 d, 1 d, 2 d, 4 d, 8 d, 16 d, and 32 d, with six replicates per sampling point. The plant roots were rinsed with deionized water, and the plant samples were divided into roots, stems, and leaves, with fresh weights recorded. The samples were inactivated at 110 °C for 0.5 h, then dried at 70 °C until reaching a constant weight, after which the dry weights were recorded. Additionally, the leaves were divided into upper, middle, and lower leaf sections, as shown in Figure S3 and Table S2. Dried samples (≤0.1 g) were fully combusted using an OX501 biological oxidizer (RJ Harvey Instruments Co., Tappan, NY, USA). The combustion chamber temperature was set at 910 °C, the catalytic chamber at 680 °C, and the combustion time was 4 min. The nitrogen and oxygen flow rates were maintained at 360–380 cc. The resulting ^14^CO_2_ from combustion was absorbed with 12 mL of scintillation cocktail II. The nutrient solution from the brown bottles and the deionized water used for root washing were mixed, and 1 mL of this mixture was added to 10 mL of scintillation cocktail I and 1 mL of ethylene glycol ether. The ^14^C radioactive activity in the plant roots, stems, leaves, and nutrient solution was measured using a TriCarb-2910 liquid scintillation counter (LSC, PerkinElmer Inc., Shelton, CT, USA).

### 2.4. Quantitative Radioautographic Imaging

After 32 days, Chinese flowering cabbage and water spinach plants exposed to carbamazepine were selected. The plants were washed with deionized water, dried with absorbent paper, and then flattened at 110 °C for fixation. The samples were wrapped with plastic wrap and put on a phosphor imaging screen, which was placed in an exposure box for one month in the dark. After exposure, the phosphor screen was scanned using a Typhoon FLA 9500 Imager (GE Healthcare Co., Chicago, IL, USA). The scanning mode was set to “phosphor” with the photomultiplier tube voltage at 500 V and a resolution of 200 μm.

### 2.5. Subcellular Distribution

Thirty milliliters of nutrient solution containing ^14^C-carbamazepine (0.18 μCi) at a concentration of 1 mg/L was added to 50 mL amber glass bottles. The amber bottles were wrapped with aluminum foil to prevent light exposure. Sampling points were set at 2, 4, 8, and 16 days, with four replicates per sampling point. After sampling, the roots were rinsed thoroughly with deionized water, and the Chinese flowering cabbage plants were separated into root, stem, and leaf parts.

The subcellular components of the cabbage were fractionated into the cell wall, plastids, nucleus, mitochondria, and soluble fraction according to the differential centrifugation method described by Chen et al. [22]. After being finely cut into different plant parts, they were mixed with pre-cooled extraction buffer at a ratio of 1:5 (m/v) and ground uniformly using a pre-cooled mortar and pestle. The homogenate was transferred to 50 mL centrifuge tubes and centrifuged at 300× *g* for 5 min in a high-speed centrifuge. The pellet containing cell wall material and cell wall fragments was collected. The supernatant was centrifuged at 1500× *g* for 10 min (for root samples, 2500× *g* for 20 min), and the resulting pellet was considered the plastid fraction (including chloroplasts, chromoplasts, and leucoplasts). The supernatant from the first centrifugation step was then centrifuged at 5000× *g* for 20 min, and the pellet was treated as the nuclear fraction. The supernatant from the second centrifugation step was centrifuged at 15,000× *g* for 30 min, and the pellet was considered the mitochondrial fraction. The final supernatant was regarded as the soluble fraction. All procedures were conducted at 4 °C. The separated cell wall and organelle fractions were freeze-dried to a constant weight and weighed, then combusted using a biological oxidizer, and their ^14^C radioactivity was measured using scintillation cocktail II. One milliliter of the soluble fraction was mixed with 10 mL of scintillation cocktail I and 1 mL of ethylene glycol ether, and the ^14^C radioactivity was measured on an LSC.

### 2.6. Safranin O and Fast Green Staining

The microstructure of plant tissues grown in hydroponic systems was observed through transverse sectioning, following the method outlined by Liu et al. [23]. Plant samples were fixed in 70% FAA solution overnight, and then root tissues were dehydrated using a series of alcohols and tertiary butyl alcohol, followed by infiltration with paraffin at 42 °C and 52 °C, respectively. The tissues were then embedded in paraffin at 52 °C and sectioned using a rotary microtome. Transverse sections, 0.01 mm thick, were stained with Fast Green. The sections were examined using a PANNORAMIC MIDI (3DHISTECH Ltd., Budapest, Hungary), and images were captured with SlideViewer software (3DHISTECH Ltd., V2.5).

### 2.7. Data Analysis

All data are presented as mean ± standard error (mean ± SE). Figures were processed and plotted using OriginPro 9.0.0 software, and statistical analyses were performed using SPSS 19.0 software. The stained tissue areas were quantified by using ImageJ software 1.54f.

## 3. Results and Discussion

### 3.1. Mass Balance in the Water-Plant System

**Figure 1:**
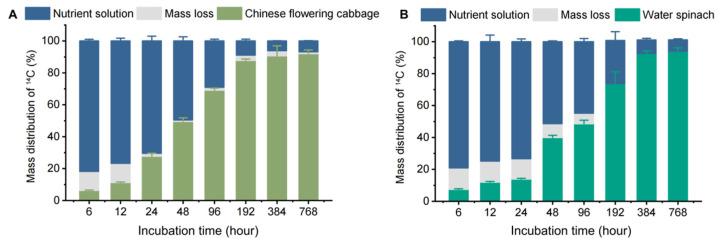
The ^14^C mass balance of ^14^C-carbamazepine and its metabolites in hydroponic systems: (**A**) denotes the Chinese flowering cabbage hydroponic system, where mass loss refers to the ^14^C portion not detected in the system, and (**B**) corresponds to the water spinach hydroponic system. Error bars represent the standard deviation (*n* = 6).

To investigate the uptake, accumulation, and removal of carbamazepine in a hydroponic system with Chinese flowering cabbage and water spinach, our experiment divided the entire cultivation system into the following three parts: the plant, the water, and the “loss” section. Figure 1 shows the distribution of ^14^C-carbamazepine and its metabolites, demonstrating that both plant species can absorb and accumulate carbamazepine from the water.

The percentage of ^14^C in the water decreased over time, while uptake by plants increased, with both species gradually accumulating ^14^C. In the first 196 h, Chinese flowering cabbage absorbed more ^14^C than water spinach; by 384 and 768 h, uptake differences between the two species were not statistically significant (*p* > 0.05). After 768 h, 92.0% and 94.1% of ^14^C-carbamazepine and its metabolites were found in Chinese flowering cabbage and water spinach, respectively, with only 6.77% and 7.06% remaining in the water. Mass balance analysis showed ^14^C recovery rates ranging from 88.0% to 99.1% in Chinese flowering cabbage and 86.6% to 101% in water spinach. In contrast, the ^14^C recovery rate was only around 50% in hydroponic systems with ^14^C-caffeine and ^14^C-benzene kresoxim-methyl, with the “loss” section being mineralized into carbon dioxide within the system [22,24]. In this study, we used brown glass containers and foil wrapping to minimize photodegradation, suggesting any “loss” was likely due to microbial metabolism in water or plant metabolites. Consistent with our findings, Li et al. [13] reported minimal mineralization of carbamazepine in soil and no carbon dioxide and volatile emissions in a soil–plant system, suggesting carbamazepine’s stability in plant systems.

The uptake and transport of organic pollutants in plants depend on pollutant properties (e.g., p*K*_a_, log *K*_OW_, and molecular size), water or soil characteristics (e.g., pH, temperature, and organic content), plant species, and environmental factors [25]. The 6.77–7.06% carbamazepine remaining in water after 768 h suggests that variability in pollutant absorption could be influenced by both the chemical properties of pollutants and plant physiology. The results showed that Chinese flowering cabbage and water spinach removed carbamazepine with efficiencies of 93.2% and 93.0%, respectively, surpassing previously reported values of 34.0–82.0% in other plants, including *Lolium perenne*, *Typha* spp., Iris sibirica, *Zantedeschia aethiopica*, and *Scirpus validus* [26,27,28]. Different plants exhibit varying pollutant absorption abilities. In this study, water spinach displayed higher removal efficiency for carbamazepine, suggesting its potential effectiveness as a phytoremediation agent. These findings contribute valuable insights to phytoremediation research and organic pollutant removal in environmental systems.

### 3.2. Distribution and Accumulation Trend of Carbamazepine in Plant Organs

**Figure 2:**
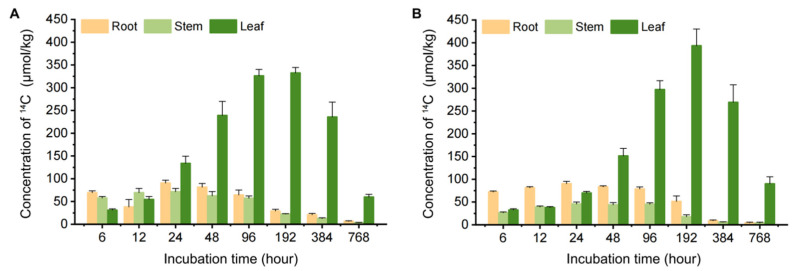
^14^C concentration dynamics in different tissues of Chinese flowering cabbage and water spinach: (**A**) Chinese flowering cabbage system with ^14^C-carbamazepine introduced and (**B**) water spinach system with ^14^C-carbamazepine introduced. Error bars represent the standard deviation (*n* = 6).

In the hydroponic vegetable system, Chinese flowering cabbage and water spinach effectively absorbed and accumulated carbamazepine from the water. Therefore, the distribution of ^14^C-carbamazepine and its metabolites within the roots, stems, and leaves of these plants was quantified (Figure 2). Over the 32-day cultivation period, carbamazepine and its metabolites were absorbed by the roots and subsequently transported to the aboveground edible parts.

In the Chinese flowering cabbage system, the ^14^C concentration in leaves and stems initially increased and then declined, while in roots, it decreased, briefly increased, and then decreased continuously. At 768 h, the ^14^C concentrations in roots, stems, and leaves were 6.69 μmol/kg, 3.61 μmol/kg, and 60.0 μmol/kg, respectively. In water spinach, the ^14^C concentrations in all parts showed an initial increase followed by a decrease, reaching 4.56 μmol/kg in roots, 4.05 μmol/kg in stems, and 90.3 μmol/kg in leaves at 768 h. At the final sampling point, the ^14^C-carbamazepine and its metabolite levels in leaves were 9–22 times higher than in roots and stems, indicating a strong tendency for leaf accumulation. Previous research has demonstrated that plants, including radish, ryegrass, lettuce, spinach, cucumber, celery, and pepper, can absorb and accumulate carbamazepine, with leaf concentrations ranging from 2.90 to 67.0 ng/g [27,29,30]. Studies by Tanoue et al. [31] revealed that moderately polar compounds like carbamazepine tend to transport to aerial parts, aligning with our findings.

The uptake of organic pollutants by plants is typically considered a continuous partitioning process between water-root and root-stem-leaf phases [32]. In this study, as roots were directly exposed to polluted water, carbamazepine absorption by roots occurred prior to stems and leaves, leading to peak concentrations in roots before the aboveground parts. The subsequent decline in pollutant concentrations across all plants contributed to factors such as growth dilution, transport, and metabolism [33,34]. During early cultivation, slower plant growth allowed for greater ^14^C accumulation; however, as biomass increased, dilution effects became more pronounced. Additionally, pollutants absorbed by roots were further transported to stems and leaves and likely underwent metabolic degradation, contributing to the observed initial rise and later decrease in concentrations. Research suggests that carbamazepine can be metabolized in plants, yielding 10, 11-epoxide-carbamazepine and 10, 11-dihydroxy-carbamazepine, and plant species have a greater effect on this metabolism than soil properties or application methods, with tomato and wheat often exhibiting more extensive metabolism than lettuce [35]. Epoxide-carbamazepine may be as or more biologically active than the parent compound, underscoring the need to distinguish these metabolites for accurate risk assessment [36]. In our hydroponic study on Chinese flowering cabbage and water spinach, we measured a total of ^14^C-labeled carbamazepine (including its metabolites) rather than isolating each metabolite. Nonetheless, our findings highlight the importance of accounting for both parent and metabolite forms when evaluating the environmental and dietary impacts of pharmaceutical contaminants in crops. Overall, these distribution patterns in plant tissues reflect various influences, including pollutant transport pathways, root affinity, and exposure duration.

Notably, ecotoxicological studies have shown that carbamazepine exhibits acute toxicity at environmental concentrations, and high doses may pose teratogenic risks [15]. According to Ghannoum et al., an intake exceeding 100 mg/kg requires emergency treatment; we therefore used this level as a reference threshold for daily carbamazepine intake [37]. We assessed the potential human health risk by calculating daily exposure from consuming Chinese flowering cabbage and water spinach, following the formula of Chen et al. [22]. The resulting values, ranging from 0.59 to 0.74 mg/kg, fall well below the reference limit, indicating minimal health risks under the conditions of our study.

### 3.3. Distribution of Carbamazepine in Different Leaf Sections

**Figure 3:**
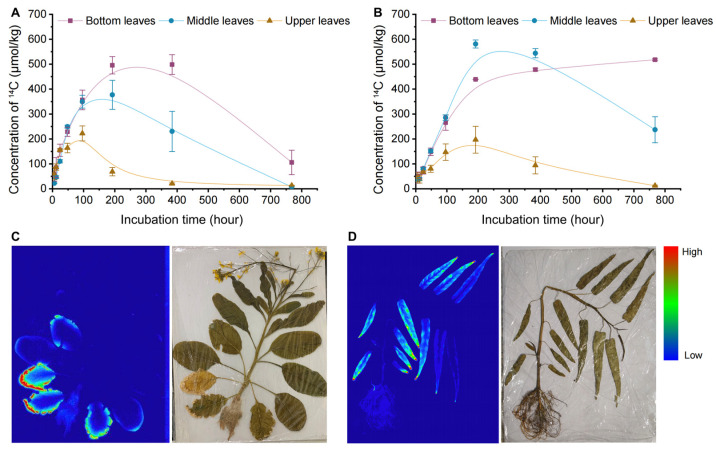
Distribution and accumulation of ^14^C-carbamazepine in plants: (**A**,**B**) dynamic distribution of ^14^C-carbamazepine in various leaf sections of Chinese flowering cabbage and water spinach. (**A**) represents the Chinese flowering cabbage system, and (**B**) represents the water spinach system. (**C**,**D**) Radioautographic imaging of ^14^C-carbamazepine distributed in plants. (**C**) shows Chinese flowering cabbage, and (**D**) shows water spinach. Blue serves as the background color, with higher brightness indicating greater ^14^C content. Error bars represent the standard deviation (*n* = 6).

To clarify the distribution of carbamazepine in the edible parts of Chinese flowering cabbage and water spinach, we divided their leaves into bottom, middle, and upper sections according to growth stage. Figure 3A,B illustrates the dynamic concentrations of ^14^C-carbamazepine and its metabolites in different leaf sections. At 6 h, ^14^C concentrations in bottom, middle, and upper leaves of Chinese flowering cabbage were 57.8, 22.1, and 60.9 μmol/kg, respectively, while in water spinach, they were 44.8, 36.0, and 46.1 μmol/kg. Concentrations in all parts of Chinese flowering cabbage and the middle and upper leaves of water spinach peaked between 48 and 384 h, subsequently declining, whereas ^14^C levels in the basal leaves of water spinach stabilized and accumulated, with concentrations at 768 h reaching 518 μmol/kg, compared to 106 μmol/kg in Chinese flowering cabbage. When the concentration of carbamazepine in the soil is less than 5 mg kg^−1^, the concentration of carbamazepine in new zucchini leaves is approximately 1.6 times higher than that in older leaves. However, when the soil concentration of carbamazepine exceeds 5 mg kg^−1^, the concentration in old leaves is higher than in new leaves [10]. Shenker et al. [38] demonstrated that plant uptake of carbamazepine may occur through passive transport, making it unrestricted by the root membrane. Additionally, the relatively low hydrophobicity of carbamazepine enables it to be transported via the transpiration stream from the roots and accumulate in mature and older leaves. This suggests that the distribution of carbamazepine in plants is also influenced by cultivation methods and plant species.

Substance transport in plants occurs via the vascular system, where xylem, composed of continuous hollow tubes, offers minimal resistance to liquid flow. Organic pollutants, absorbed by roots, are transported along the xylem to aboveground parts with water flow [19]. For leafy vegetables with a growth cycle of approximately 35 days, continuously growing leaves function as both assimilation and nutrient storage organs. In later growth stages, nutrients primarily accumulate in the larger, more mature leaves. As growth progresses, early-developed leaves transition to lower positions, while new, tender leaves emerge at the top. The lower leaves, being larger, receive more transpired water than the middle or upper leaves. Additionally, declining carbamazepine concentrations in water over time contributed to the reduced accumulation in younger leaves, which was consistent with the ^14^C radioactivity images in the vegetable leaves.

### 3.4. Quantitative Radioautographic Imaging of ^14^C-Carbamazepine

To visually assess the uptake and accumulation of carbamazepine in plants, autoradiography was used to track ^14^C distribution in plants at the final sampling point. Autoradiographs show that ^14^C-carbamazepine and its metabolites primarily accumulate in the leaves of Chinese flowering cabbage and water spinach, particularly in the lower leaves (Figure 3C,D). After root uptake, ^14^C is transported upward via the xylem, then distributed unevenly within the leaves, concentrating mainly at the edges and tips of the lower leaves. In plants, transpiration flow transports the three organic pollutants from the water to the aboveground parts, where water evaporates from the leaves through stomata into the surrounding atmosphere. The absorbed organic pollutants are continuously transported to the leaf edges via the transpiration stream, while transpiration from the petioles is minimal [39]. Additionally, due to the longer growth period and lower water potential pressure, the transpiration water volume is higher in the lower leaves. The concentration of organic pollutants in the water decreases over time, and as the same volume of transpiration water passes through the plant, the amount of organic pollutants absorbed gradually decreases. Ultimately, after prolonged exposure, more ^14^C was detected at the edges of the lower leaves.

Studies suggest that organic pollutants taken up by roots may either bind to root lipids or pass through the Casparian strip into the endodermis, reaching tracheid and xylem tissues [20]. Transpiration subsequently transports these pollutants to aboveground tissues, ultimately distributing them in stem and leaf tissues [19]. Water-soluble pollutants generally have reduced permeability through the Casparian strip, yet migrate more readily through the plant once inside. Wu et al. [40] found that moderately polar compounds are easily transported to the aboveground parts, while Chen et al. [24] showed that the lipophilic compounds are primarily retained in rapeseed roots, with minimal translocation to stems and leaves.

### 3.5. Subcellular Distribution of Carbamazepine in Chinese Flowering Cabbage Cells

**Figure 4:**
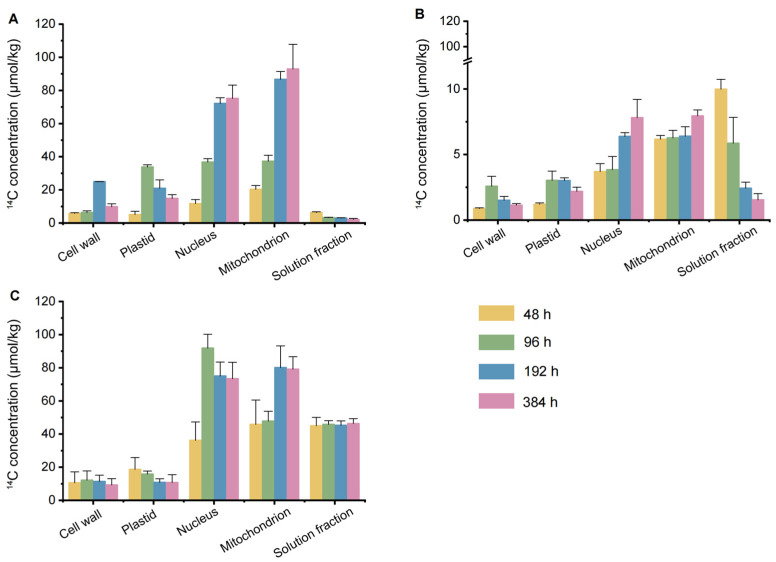
Subcellular distribution of ^14^C-carbamazepine and its metabolites in the roots, stems, and leaves of Chinese flowering cabbage: (**A**) roots, (**B**) stems, and (**C**) leaves. Error bars represent the standard deviation (*n* = 6).

The subcellular distribution of ^14^C-carbamazepine and its metabolites in the root, stem, and leaf cells of Chinese flowering cabbage is shown in Figure 4, indicating their presence across the cell wall, plastids, nucleus, mitochondria, and soluble fractions. This distribution demonstrates that carbamazepine and its metabolites can permeate the root cell wall and membrane, subsequently translocating to aboveground tissues.

In this study, ^14^C-carbamazepine and its metabolites were primarily localized in the roots and leaves, with minimal concentrations in the stem. In root cells, ^14^C initially increased and then decreased in the cell wall and plastids, while it steadily increased in the nucleus and mitochondria and declined in the soluble fraction. The overall distribution trend in root cells was mitochondria (20.3–92.9 μmol/kg) > nucleus (11.7–75.2 μmol/kg) > plastids (5.06–33.8 μmol/kg) ≈ cell wall (5.77–24.9 μmol/kg) > soluble fraction (2.13–6.30 μmol/kg). Over time, carbamazepine and its metabolites decreased in the root cell wall, plastids, and soluble fractions, gradually accumulating in the nucleus and mitochondria. Upon reaching the stem, the concentration of ^14^C-carbamazepine and its metabolites was significantly lower than in the roots, with the distribution trend in stem cells showing a pattern of soluble fraction (1.54–9.98 μmol/kg) > mitochondria (6.17–7.95 μmol/kg) > nucleus (3.70–7.81 μmol/kg) > plastids (1.20–3.02 μmol/kg) > cell wall (0.88–2.58 μmol/kg). This suggests that transpiration flow facilitates the transport of carbamazepine and its metabolites from the stem to the leaves. By the late cultivation stage, ^14^C-carbamazepine and its metabolites were primarily concentrated in the leaf nucleus (36.2–75.0 μmol/kg), mitochondria (45.9–80.1 μmol/kg), and soluble fractions (45.0–46.3 μmol/kg), significantly higher than other subcellular components (*p* < 0.05). The concentrations in the cell wall and plastids of leaf cells decreased over time, while the soluble fraction remained stable.

The distribution of organic pollutants in plant cells is closely linked to cellular components. The soluble fraction, primarily vacuolar sap and ribosomes, acts as a cellular buffer containing cytosol and other water-soluble substances, with vacuoles serving as “waste depots” that play a major role in pollutant catabolism [41]. Plastids, essential organelles unique to plant cells, are involved in anabolic processes and vary as chloroplasts, chromoplasts, or leucoplasts based on pigment content. Due to centrifugation limitations, cell membranes are included in the soluble fraction in current studies [42]. Lipid-rich organelles, such as mitochondria and plastids, are primary reservoirs for lipophilic pollutants, typically containing 15–30% lipids, whereas cell walls, composed mainly of polysaccharides, contain less than 10% lipids [42,43]. This lipid-driven partitioning leads lipophilic pollutants to accumulate in organelles with triclosan (log *K*_OW_ = 4.76) and its metabolites [44]. Conversely, highly water-soluble pollutants primarily localize in the aqueous phase of plant cells, explaining the distribution of carbamazepine in Chinese flowering cabbage, where it predominantly appears in the leaf soluble fraction [45]. Li et al. [46] reported that carbamazepine and its metabolites in celery leaves were primarily in the soluble fraction (80.3%). In our study, carbamazepine in the soluble fraction of Chinese flowering cabbage leaves ranged from 93.5 to 94.7%, which is higher than that observed in celery leaves. This difference may be attributed to the shorter cultivation period and species-specific differences.

### 3.6. Influencing Factors

#### 3.6.1. Physicochemical Properties of Organic Pollutants

Plant cell membranes lack specific transport proteins for organic pollutants, meaning plant absorption of these pollutants relies primarily on diffusion, which is influenced by the compounds’ chemical properties, particularly hydrophobicity [17,47,48]. The partition coefficient (log *K*_OW_) serves as a key measure of hydrophobicity, with values between 0.5 and 3 indicating compounds that are effectively removed by plants through direct uptake, as their lipophilicity enables them to cross lipid bilayers and enter cell cytosol [49,50]. Plant uptake of many compounds is positively correlated with log *K*_OW_, though some studies, such as those by Reinhold et al. [51], indicate uptake can be independent of hydrophobicity. Highly polar compounds (log *K*_OW_ < 1) show low plant absorption [49], while more hydrophilic compounds may exhibit higher root uptake and translocation [52]. Research on constructed wetlands also shows that aquatic plants significantly improve pollutant removal compared to unplanted systems [53], with hydroponic studies demonstrating that polar compounds can be efficiently absorbed and translocated in plants, especially to shoots [54]. The root concentration factor (RCF) is commonly used to describe the capacity of a plant to accumulate a chemical from its surrounding medium, which is calculated by dividing the concentration of the chemical in the roots by its concentration in the original solution. Li et al. [55] synthesized data on 37 organic pollutants across hydroponic studies, finding a strong linear correlation (R^2^ = 0.908) between compounds with log *K*_OW_ ≥ 1.5 and log RCF, which fitted the equation log RCF (hydroponics) = 0.72 log *K*_OW_ −1.14. They further predicted that for compounds with log *K*_OW_ ≤ 1.1, the linear correlation coefficient R^2^ between log RCF and log *K*_OW_ would approach 0.9, which is consistent with the findings of Briggs et al. [56], whose fitted equation was log RCF (root immersion) = 0.77 log *K*_OW_—1.52.

**Figure 5:**
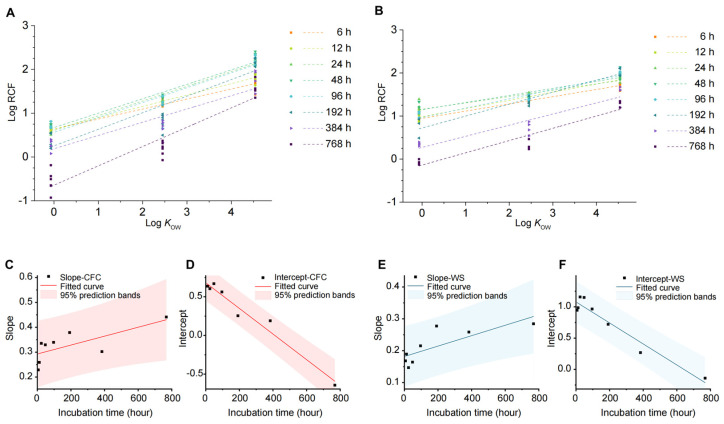
Factors influencing the uptake and transport of carbamazepine in the hydroponic vegetable system. (**A**,**B**) Relationship between root bioconcentration factor and log *K*_OW_. A for the Chinese flowering cabbage system (CFC) and B for the water spinach system (WS). (**C**–**F**) Relationship of the intercept and slope of the linear equation between root bioconcentration factor and log *K*_OW_ with cultivation time.

This study examines the relationship between the log RCF of caffeine, carbamazepine, and benzene kresoxim-methyl (log *K*_OW_ values of −0.07, 2.45, and 4.54, respectively) in Chinese flowering cabbage and water spinach. Results show a strong linear correlation (R^2^ > 0.8; Table S3) between log RCF and log *K*_OW_ across sampling points, indicating higher lipophilicity enhances root accumulation in both plants (Figure 5A,B). While hydrophobic partitioning is a primary mechanism for nonpolar compounds (e.g., PAHs and benzene) entering plants, other factors like hydrogen bonding and molecular interactions also affect polar compound uptake. Thus, log *K*_OW_ alone does not account for plant absorption; additional factors like vapor pressure, molecular weight, and environmental conditions (e.g., dissolved organic carbon, pH, organic matter, and temperature) are relevant. Chuang et al. [39] found that compounds with molecular weights below 300 g/mol efficiently translocate from roots to shoots, while those above 400 g/mol largely accumulate in the roots. This study aligns with the following observations: caffeine (194 g/mol) and carbamazepine (236 g/mol) translocate readily to shoots, while benzene kresoxim-methyl (326 g/mol) primarily accumulates in roots, illustrating molecular size effects on translocation patterns.

#### 3.6.2. Cultivation Time

Cultivation time is an important factor influencing the absorption and accumulation of organic pollutants in plants. Analysis of the linear relationship between RCF and log *K*_OW_ across eight sampling points revealed consistent linearity, though with varying slopes and intercepts. As shown in Figure 5C–F, intercepts exhibited a high linear correlation with time (R^2^ > 0.9), decreasing as cultivation time increased. This indicates a reduction in log RCF and a shift in pollutant accumulation from roots to aboveground parts. In contrast, slopes showed a weaker correlation with time (R^2^ ≈ 0.5) but increased over time, suggesting that longer cultivation enhances differences in pollutant accumulation in roots. These findings provide useful insights and data for understanding plant absorption and accumulation dynamics of organic pollutants.

#### 3.6.3. Plant Lipid Content

Plants are mainly composed of water, carbohydrates, and lipids, with varying proportions across different plant tissues. Chiou et al. [45] found that pollutants with log *K*_OW_ < 1 are absorbed primarily due to root water content, those with log *K*_OW_ ≈ 2 are influenced by both water and lipid content, while lipids dominate for log *K*_OW_ > 3 pollutants. In this study, we measured lipid, water, and carbohydrate contents in different parts of Chinese flowering cabbage and water spinach to examine their influence on pollutant absorption and distribution.

A comparison of the three pollutants’ distribution across plant tissues (Figure 2) showed that pollutant concentration peaks earlier in Chinese flowering cabbage roots (12–96 h) than in water spinach (24–192 h), suggesting faster upward translocation in the former. Higher lipid content (2.07%) in water spinach roots likely retains pollutants, reducing upward movement (Table S3). The lipophilic pollutant benzene kresoxim-methyl predominantly accumulated in roots, with leaf lipid contents being 5.97 and 2.68 times that of roots in Chinese flowering cabbage and water spinach, respectively, further explaining the higher benzene kresoxim-methyl levels in Chinese flowering cabbage shoots [24]. Similarly, Schroll et al. [57] reported that hexachlorobenzene (HCB) residues in roots increased with lipid content, supporting our findings that lipid content significantly affects pollutant retention in roots.

#### 3.6.4. Plant Xylem

Organic pollutants can be transported to shoots, leaves, or fruits through either the xylem or phloem. However, the boundary between xylem-mobile and phloem-mobile compounds is not always clear-cut. While phloem-mobile compounds can also move through the xylem, those primarily exhibiting xylem mobility can still enter the phloem [20]. For nonionized compounds, those with log *K*_OW_ < 0 are considered ambimobile, whereas those with intermediate lipophilicity (0 < log *K*_OW_ < 3) typically move only in the xylem. Acids with p*K*_a_ < 7 and log *K*_OW_ < 3 remain in the phloem due to ion-trapping mechanisms and can be transported to fruits. In contrast, bases with p*K*_a_ > 7 and log *K*_OW_ < 0 are generally ambimobile, while those with 0 < log *K*_OW_ < 4 predominantly move through the xylem [58].

**Figure 6:**
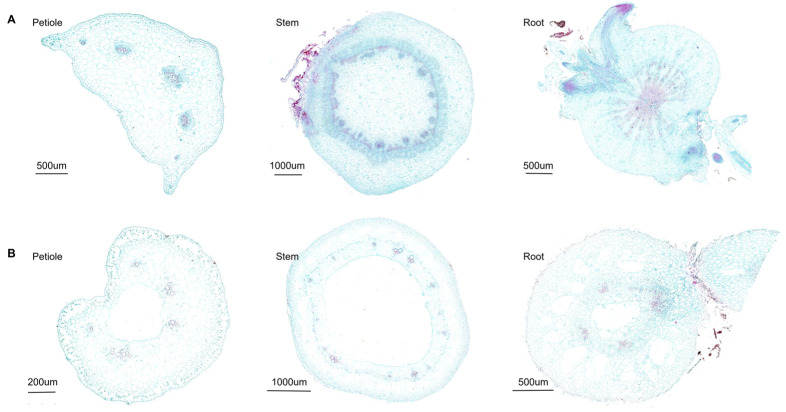
Cross-sectional diagram of Chinese flowering cabbage (**A**) and water spinach (**B**). Both Safranin O and Fast Green stain plant cell walls, with red indicating lignified and suberized cell walls and green indicating cellulose cell walls.

For carbamazepine, with a log *K*_OW_ of 2.45 and a p*K*_a_ of approximately 13.9 [59], it is more likely to be transported through the xylem according to the above theory. By examining the cross-sectional structures of roots, stems, and petioles in Chinese flowering cabbage and water spinach (Figure 6) and quantifying xylem area with ImageJ (Table S5), we found that the xylem region was notably larger in Chinese flowering cabbage than in water spinach. This more abundant and concentrated xylem structure in Chinese flowering cabbage appears to facilitate faster upward transport of carbamazepine. In comparison, water spinach has a greater amount of intercellular space, primarily for gas exchange, but this does not significantly impact the overall uptake and transport of carbamazepine. In summary, for carbamazepine, the higher the xylem content in the plant, the faster its upward transport.

## 4. Conclusions

This study examined the uptake, subcellular distribution, and accumulation of carbamazepine in two plants of Chinese flowering cabbage and water spinach. Both plants effectively took up and bioaccumulated ^14^C-labeled carbamazepine, with over 92% of the total ^14^C found in plant tissues by 768 h and less than 7% remaining in the water. Carbamazepine accumulated primarily in the lower leaves, reaching 90.3 μmol/kg, and was confirmed by autoradiography. The compound penetrated cell walls and membranes, accumulating in root organelles and the soluble fraction of leaf cells. Further analysis showed that its uptake and translocation in plants were influenced by log *K*_OW_ and lipid content, with higher lipid content in water spinach roots enhancing upward transport. Additionally, a higher xylem content in the plant accelerated carbamazepine transport. These findings help us understand the eco-environmental risks of carbamazepine and provide important insights into the behavior of other organic pollutants in plants. However, in natural soil systems, factors such as soil adsorption, microbial degradation, and competing ions can substantially influence contaminant behavior and must be considered when extending our hydroponic findings to field conditions.

## Data Availability

The data are available from the corresponding author.

## Supplementary Materials

The following supporting information can be downloaded at: https://www.mdpi.com/article/10.3390/biology14040343/s1, Figure S1: Chinese flowering cabbage seed germination (A), Chinese flowering cabbage seedlings (B), and water spinach seedlings (C); Figure S2: Hydroponic vegetables illustration (A. Chinese flowering cabbage and B. water spinach); Figure S3: Example of plant leaf number; Table S1: Physiochemical properties of carbamazepine; Table S2: Classification of leaves; Table S3: The relevant parameter of correlation between root concentration factor and log *K*_OW_; Table S4: Determined weight percent compositions of Chinese flowering cabbage and water spinach. Table S5: Xylem-stained area in different plant tissues.

## Abbreviations

The following abbreviations are used in this manuscript:

PPCPs	Pharmaceutical and Personal Care Products
CBZ	carbamazepine
POPOP	1,4-di(5-phenyloxazole-2-yl)
PPO	5-diphenyloxazole
LSC	liquid scintillation counter
RCF	root concentration factor
