# Supplementary material for: Accumulation and Subcellular Distribution Patterns of Carbamazepine in Hydroponic Vegetables
Source: Biology (Basel). 2025 Mar 26;14(4):343. doi: 10.3390/biology14040343 (PMC12024843; doi:10.3390/biology14040343)
Supplement: Supplementary file 1 [file biology-14-00343-s001.zip › biology-3541498-supplementary.pdf]

Supplementary Materials for

Accumulation and Subcellular Distribution Patterns of Carbamazepine in Hydroponic Vegetables

Sihan Yao, Yan Chen, Nan Zheng, Ting Chen, Sufeng Zhang, Zhiyang Yu, Haiyan Wang

Corresponding author: Haiyan Wang, wanghaiyan@zju.edu.cn

This file includes:

Fig. S1 to S3

Table S1 to S5

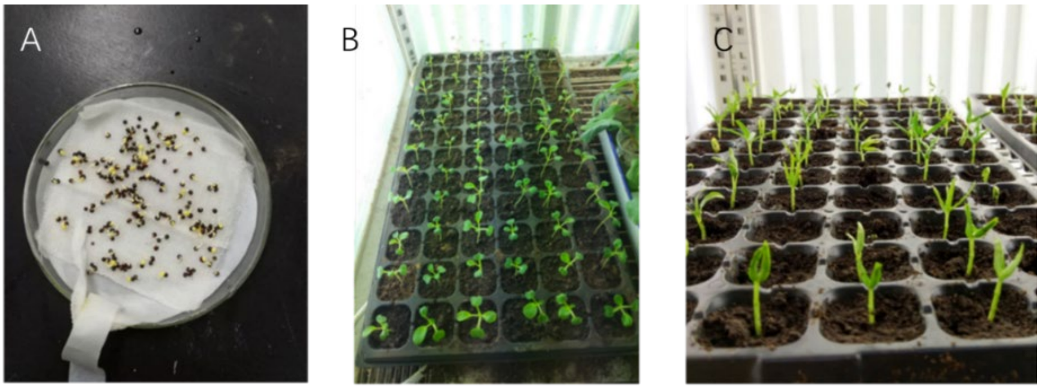

Figure S1 Chinese flowering cabbage seed germination (A), Chinese flowering cabbage seedlings (B), and water spinach seedlings (C).

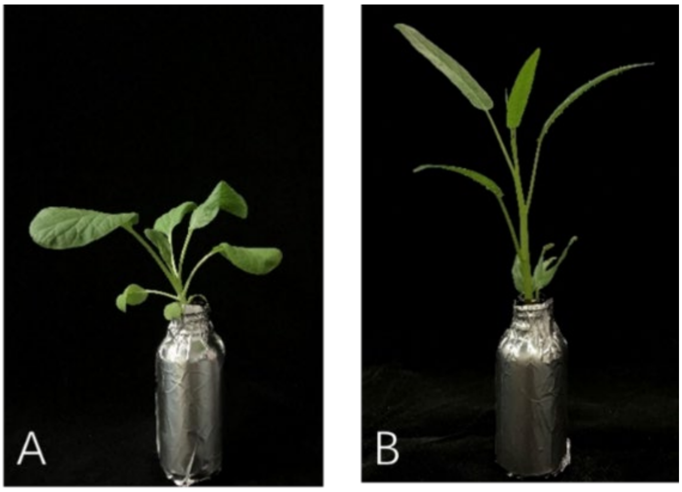

Figure S2 Hydroponic vegetables illustration (A. Chinese flowering cabbage and B. water spinach).

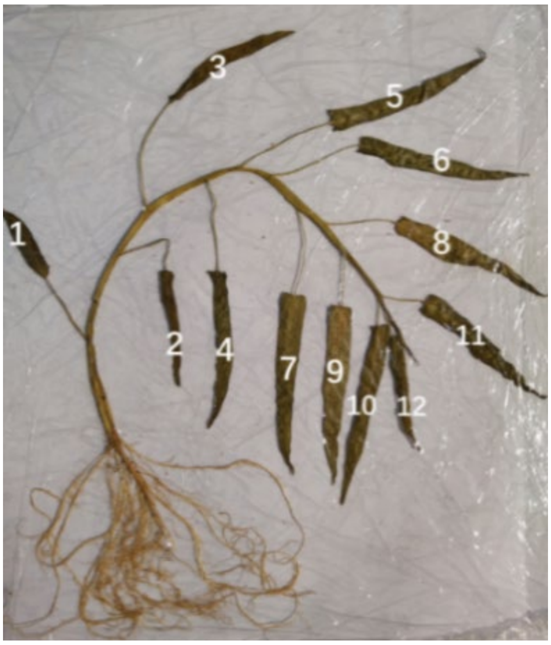

Figure S3 Example of plant leaf number.

Table S1 The physiochemical properties of carbamazepine.

Property	Carbamazepine
Molecular structure	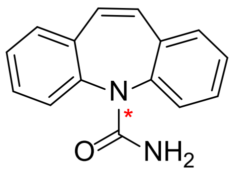
Molecular formula	$C_{15}H_{12}N_2O$
Relative molecular mass	236.27
log K_{OW}	2.45
Water solubility (mg L^{-1}) [℃]	18 [25℃]
Boiling point (℃)	399.6 ± 45.0

Note: The asterisk (*) indicates the ^{14}C labeling site.

Table S2 Classification of plant leaves.

Number of plant leaves	Classification		
	Upper leaves	Middle leaves	Bottom leaves
5	2	2	1
6	2	2	2
7	3	2	2
8	3	3	2
9	3	3	3
10	4	3	3
11	4	4	3
12	4	4	4
13	5	4	4
14	5	5	4
15	5	5	5
16	6	5	5
17	6	6	5
18	6	6	6
19	7	6	6
20	7	7	6
21	7	7	7

Note: The leaves of all plant samples are classified as upper, middle, and bottom leaves. As shown in Fig. S3, leaves are numbered from bottom to top. For example, when a plant has 12 leaves, leaves 1 to 4 are considered lower leaves, leaves 5 to 8 are middle leaves, and leaves 9 to 12 are upper leaves.

Table S3 The relevant parameter of correlation between root concentration factor and log K_{OW}.

Plant	Coefficient of determination	6 h	12 h	24 h	48 h	96 h	192 h	384 h	768 h
Chinese flowering cabbage	R^2	0.9632	0.9779	0.9719	0.9203	0.8472	0.8506	0.8095	0.8895
Water spinach		0.9766	0.9396	0.8721	0.8441	0.9307	0.9272	0.8686	0.9016

Table S4 Weight percentage compositions of Chinese flowering cabbage and water spinach.

Plant	Tissue	Lipids (%)	Water (%)	Carbohydrates (%)
Water spinach	Root	2.07 ± 0.001 d	87.7 ± 0.012 e	9.96 ± 0.006 b
	Stem	2.61 ± 0.000 c	90.9 ± 0.011 c	7.33 ± 0.004 c
	Leaf	5.56 ± 0.002 a	89.3 ± 0.008 d	5.17 ± 0.005 e
Chinese flowering cabbage	Root	0.75 ± 0.001 f	88.1 ± 0.016 e	10.4 ± 0.004 a
	Stem	1.47 ± 0.000 e	93.2 ± 0.011 a	5.58 ± 0.004 d
	Leaf	4.48 ± 0.000 b	92.5 ± 0.012 b	3.44 ± 0.011 f

Note: a−f indicate significant differences for the same substance between different plant tissues ($p < 0.05$).

Table S5 Xylem-stained area in different plant tissues.

Plant species	Tissue	Xylem-stained area (μm^2)
Chinese flowering cabbage	Petiole	35685.13
	Stem	261718.75
	Root	63350.76
Water spinach	Petiole	4131.25
	Stem	9486.79
	Root	19084.64

Note: Representative cross-sectional images are shown in Fig. 6, where red staining indicates xylem regions.
